# First Dinosaurs from Saudi Arabia

**DOI:** 10.1371/journal.pone.0084041

**Published:** 2013-12-26

**Authors:** Benjamin P. Kear, Thomas H. Rich, Patricia Vickers-Rich, Mohammed A. Ali, Yahya A. Al-Mufarreh, Adel H. Matari, Abdu M. Al-Massari, Abdulaziz H. Nasser, Yousry Attia, Mohammed A. Halawani

**Affiliations:** 1 Department of Earth Sciences, Uppsala University, Uppsala, Sweden; 2 Palaeontology Department, Museum Victoria, Melbourne, Victoria, Australia; 3 School of Geosciences, Monash University, Melbourne, Victoria, Australia; 4 Saudi Geological Survey, Jeddah, Kingdom of Saudi Arabia; State Natural History Museum, Germany

## Abstract

Dinosaur remains from the Arabian subcontinent are exceedingly rare, and those that have been documented manifest indeterminate affinities. Consequently the discovery of a small, but diagnostic, accumulation of elements from Campanian-Maastrichtian (∼75 Ma) deposits in northwestern Saudi Arabia is significant because it constitutes the first taxonomically identifiable dinosaur material described from the Arabian Peninsula. The fossils include a series of possible lithostrotian titanosaur caudal vertebrae, and some isolated theropod marginal teeth that share unique character states and metric parameters (analyzed using multivariate statistical methods) with derived abelisaurids – this is the first justifiable example of a non-avian carnivorous dinosaur clade from Arabia. The recognition of titanosaurians and abelisaurids from Saudi Arabia extends the palaeogeographical range of these groups along the entire northern Gondwanan margin during the latest Cretaceous. Moreover, given the extreme paucity of coeval occurrences elsewhere, the Saudi Arabian fossils provide a tantalizing glimpse into dinosaurian assemblage diversity within the region.

## Introduction

Dinosaur fossils are extremely scarce in the Arabian Peninsula and Levant region of the Middle East. Published occurrences include isolated teeth and bones of Cretaceous brachiosaurid (Neocomian) and titanosaurian (Maastrichtian) sauropods from Lebanon [1] and Jordan [2] respectively, indeterminate sauropod limb material from Oman (Maastrichtian [3]), large theropod postcranial elements from Oman (Maastrichtian [4]) and Syria (Cenomanian or Turonian/Senonian [5]), and fragmentary ornithopod (Maastrichtian) remains from Oman [3] and Jordan [6]. The partial skeleton of an enantiornithine bird has also been documented from the Late Cretaceous of Lebanon (Cenomanian [7]), together with feather inclusions in amber from the Early Cretaceous (Neocomian) of Lebanon [8] and Jordan [9]. Jacobs et al. [10] and Schulp et al. [11] provided accounts of both undefined sauropod body fossils, and sauropod and ornithopod footprints from Jurassic-Cretaceous (Bathonian-Berriasian) strata in Yemen. Avnimelech [12] additionally described Late Cretaceous (Cenomanian) theropod tracks near Jerusalem.

**Figure 1:**
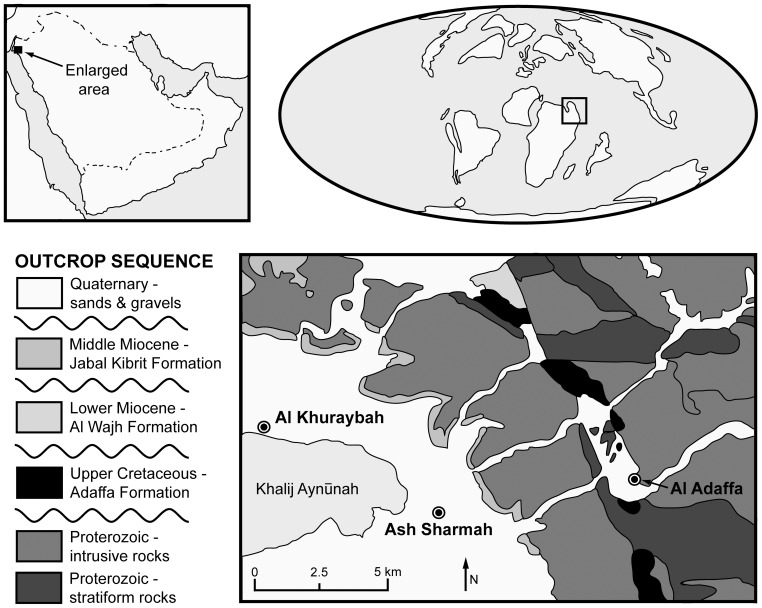
Locality maps. Simplified regional map (top left) with enlargement (bottom) showing distribution of Adaffa Formation outcrops and geographical positioning of the Arabian Peninsula during the Campanian-Maastrichtian (top right). Geological map simplified from [17] with stratigraphic terminology following [13].

Virtually nothing has been reported on dinosaurs from Saudi Arabia. Hughes and Johnson ([13] p. 59, Fig. 11 and in text on p. 60) briefly mentioned a confidential Saudi Aramco report (“Milner, A., N. Morris and P. Jeffery. 1993. *Report on Macrofossils from the Kingdom of Saudi Arabia*. Natural History Museum, London, Confidential report for Saudi Aramco”) that identified bone fragments of a “sauropod dinosaur, possibly a titanosaurid” from the Adaffa Formation, an Upper Cretaceous unit that crops out in the Midyan Peninsula region along the northeastern coast of the Red Sea (Fig. 1). Grainger ([14] p. 153) also noted some additional “tentatively confirmed” dinosaur bones, together with other vertebrate remains, collected from the Adaffa Formation in 2004–2008 by a joint team from the Saudi Geological Survey (SGS) and Egyptian Geological Museum. Subsequent appraisal of this material by Kear et al. [15], [16] documented a primarily marine fauna incorporating: indeterminate anacoracid? sharks; actinopterygians − lepisosteids, pycnodontiforms, pachycormids (cf. *Protosphyraena*) and teleosts (cf. *Enchodus* sp.); ceratodont lungfish (*Ceratodus* sp.); bothremydid turtles; dyrosaurid crocodilians; an elasmosaurid plesiosaur; plioplatecarpine mosasaurs and the widespread mosasaurine *Prognathodon*; as well as a small aquatic varanoid (cf. *Pachyvaranus*). A few dinosaur bones and teeth were also recovered from the deposit and are presented in this paper. These fossils are important because they represent the first definitive dinosaurian remains described from the Kingdom of Saudi Arabia.

No permits were required for the described study, which complied with all relevant regulations.

### Geological Setting

The Adaffa Formation is a thick sequence of cross-bedded, quartz arenite sandstones with basal conglomerates, and upper-most thin marl, siltstone, and fine-grained sandstone layers. It forms the lower-most unit of the Late Cretaceous to Paleogene Suqah Group, a series of pre-Red Sea rift strata that unconformably overly Paleozoic basement rocks in the Usfan region (Jeddah Basin) near Jeddah, and northwards into the Midyan region (Ifal Basin) of far northwestern Saudi Arabia [13]. Detailed stratigraphic assessments [13], [17] and palynological studies indicate an early Campanian−early Maastrichtian age [18].

The Adaffa Formation vertebrate macrofossils occur in two restricted graben structures, the Aynunah and Sharmah troughs. The individual elements are disarticulated and randomly distributed through thin limonitic beds near the top of the main sandstone sequence. The bone surfaces exhibit extensive surface abrasion (e.g. edge rounding and decortication) consistent with damage by wave action and/or currents prior to burial. This accords with the inferred supratidal marine to fluviatile depositional settings, with coarse clastic input from braided river outlets fed by periodic flash floods [18].

## Results and Discussion

Seven caudal vertebrae from a sauropod (SGS 0188, SGS 0213, SGS 0342, SGS 0366, SGS 0422, plus two additional unregistered fragments), and two theropod marginal teeth (SGS 0061, SGS 0090), were recovered during an exhaustive excavation of a small limonitic sandstone exposure (∼10 m^2^) within the Aynunah Trough, about 11 km northeast of Al Khuraybah ([Fig. 1]; see Hughes and Johnson ([13] p. 60, Fig. 12 for a photograph of the site). These elements were found intermixed with other vertebrate remnants and numerous wood fragments, presumably sorted by turbulent water action. There was no obvious association between individual skeletal components; although, compatible anatomical positioning, size, ontogentic stage, and taxonomic affinities of at least the dinosaur material suggests derivation from single animals. All of the specimens were accessioned into the Paleontological Collection of the Saudi Geological Survey, Jeddah, Kingdom of Saudi Arabia.

**Figure 2:**
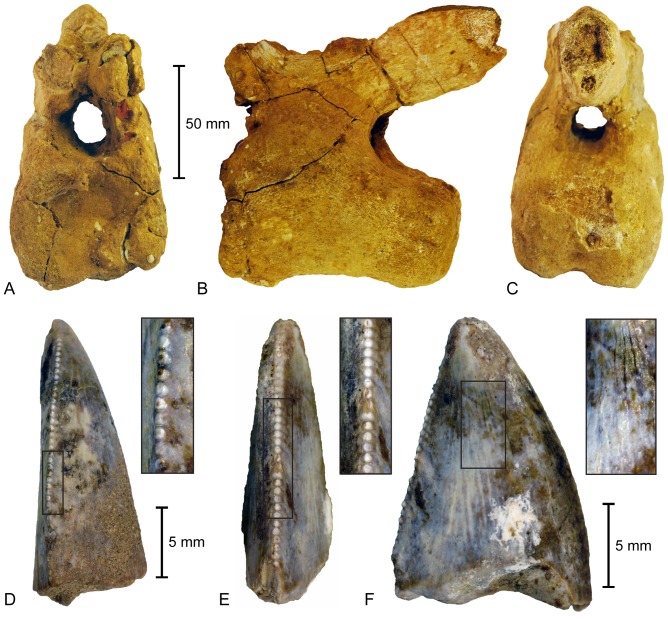
Dinosaur remains from the Adaffa Formation of Saudi Arabia. Titanosaurian distal caudal vertebra (SGS 0366) in: A, anterior; B, lateral; and C posterior views. Abelisaurid teeth including: D, crown fragment (SGS 0061) with enlargement of the distal denticles; and maxillary-dentary tooth (SGS 0090) shown in distal (E) and lateral (F) views with enlargements of the distal carina and baso-apical enamel ridges.

The sauropod vertebrae appear to form a continuous series from the posterior-distal caudal region. Unfortunately, most of the bones are badly weathered and comprise only broken parts of the centra. However, one specimen (SGS 0366 [Fig. 2A−C]) is relatively complete and retains a neural arch. Dimensions of SGS 0366 are: centrum length  =  105 mm; centrum width across the anterior articular surface  =  66 mm; lateral height of the anterior articular surface  =  56.5 mm; maximum vertebral height including neural arch  =  133 mm. The recovered centra are all cylindrical in outline and clearly procoelous, a classic feature of titanosaurians [19]. Where discernible, the ventral surface is flat and exhibits raised areas on both the anterior and posterior ends for accommodation of the chevron facets. The neural arch is anteriorly positioned (compatible with titanosauriforms [20], [21]) and the prezygapophyses, although broken and heavily weathered, would have projected anterodorsally. The spinoprezygapophyseal laminae are not fused and the prespinal lamina is present as a low, near horizontal ridge. The neural spine is elongate and posteriorly inclined reminiscent of titanosaurian taxa such as *Isisaurus* and *Neuquensaurus* [22]. The postzygaphophyses are weakly delineated.

Both of the Adaffa Formation theropod teeth are incomplete: SGS 0061 consists of only a sheared sliver, preserving parts of the lateral and distal surfaces, the latter bearing a denticle row [Fig. 2D]; SGS 0090 is a relatively complete crown with worn apex and broken base [Fig. 2E, F]. The tooth outline is narrow compared to its length and height (SGS 0090 crown base width [CBW]  =  4.7 mm; crown base length [CBL]  =  11.6; crown height [CH]  =  17.1 mm; CBW/CBL [CBR]  =  0.41). It is also low in profile (CH/CBL [CHR]  =  1.47), and nearly triangular in lateral view with the apex positioned close to the centre of the crown base (crown angle [CA]  =  82°). The gently convex mesial face is rounded and lacks a carina. Conversely, the distal profile is clearly linear, consistent with the distinctive maxillary-dentary teeth of abelisaurid ceratosaurians [23]. The distal carina is straight and comprises labio-lingually broad denticles (average density [DAVG]  =  10.9/5 mm) with basally angled interdental sulci (resembling derived carnotaurines [23], [24]). Both the labial and lingual tooth surfaces bear apically converging longitudinal ridges, which are more pronounced distally and occasionally branch towards the base [Fig. 2F]. Similar vertical enamel ridging or fluting has been reported in dromaeosaurids [25], spinosaurids [26], and ceratosaurians [27] including the abelisauroid *Masiakasaurus* [28].

### Analysis

The Adaffa Formation dinosaur remains are fragmentary but can be unambiguously referred to typical Late Cretaceous Gondwanan lineages based on discrete phylogenetic character states: Titanosauria [29] and Lithostrotia [21], diagnosed by the presence of procoelous caudal centra; and Abelisauridae, characterized by a centrally positioned tooth apex with strongly curved mesial, and straight distal profiles [30]. Baso-apically trending ridges and interdental sulci can also be variably developed in derived forms [23]. However, because some qualitative theropod tooth characters are known to be phylogenetically ambiguous [31], we conducted a series of morphometric analyses to corroborate our hypothesized affinity for the Adaffa Formation specimens, and to test their proportional similarities relative to other non-avian theropods. Measurements of SGS 0090 and SGS 0061 [Table 1] were added to the most taxon-rich matrix of theropod dental metrics obtainable from the literature [32] with taxonomic modifications introduced by Smith and Lamanna [24]. Unfortunately, a compatible metric data set was not available for sauropod postcranial elements, preventing quantitative evaluation of affinities; primary compilation of such information from original fossils and/or the literature was also beyond the scope of this paper.

**Table 1:** Measurements of SGS 0061 and SGS 0090 used in the morphometric analyses (CBL, CBW, CH, AL are in mm).

Specimen	CBL	CBW	CH	AL	CBR	CHR	CA	DA	DC	DB	DAVG
SGS 0061	-	-	19.7	-	-	-	-	12	11.2	12	11.73
SGS 0090	11.6	4.7	17.1	18.5	0.41	1.47	82	12	10.3	10.5	10.9

Parameters follow Smith et al. [32]. Abbreviations: CBL, crown base mesial-distal length; CBW, crown base labio-lingual width; CH, crown height from apex to distal enamel base; AL, apical length from medial enamel base; CBR, crown base ratio CBW/CBL; CHR, crown height ratio CH/CBL; CA, crown angle from mesial base to crown; DA, number of denticles/5 mm ( =  density) at the apical section of the distal carina; DC, distal denticle density at the mid-crown; DB, distal denticle density at the crown base; DAVG, average distal denticle density/5 mm.

**Figure 3:**
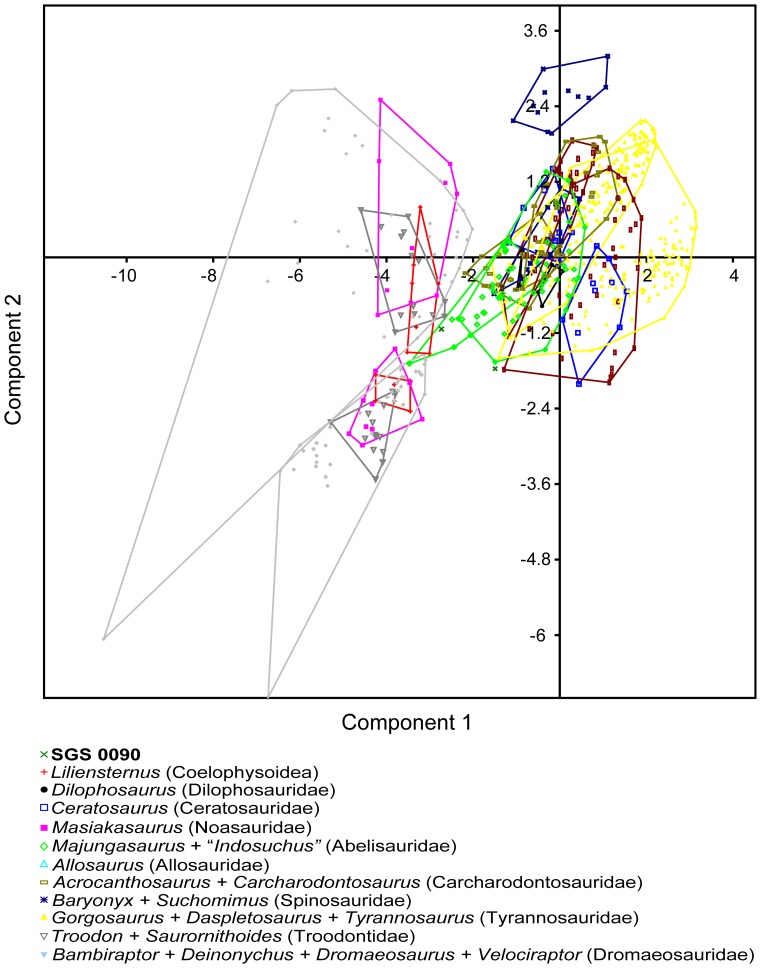
Principal components analysis plot of the most complete Adaffa Formation theropod tooth (SGS 0090) using all metric parameters. Result derived by adding SGS 0090 (green x) to the dental morphometric matrix of Smith et al. [32]. Samples were organized into 10 categories representing theropod family-level clades (see legend). Component axes 1 and 2 depict maximum discrimination in the data set; minimum scatter areas for each group indicated by polygons coded by taxon/sample colour.

We log_10_-transformed (see rationalization in Samman et al. [33] and references therein) the theropod tooth measurements and subjected them to a series of multivariate statistical analyses designed to best accommodate our small sample size and its missing information: (1) a Principal components analysis (PCA), bivariate plots, and a Discriminant analysis (DA) focusing on size correlated variables (CBL, CBW, CH, AL); (2) a parametric Multivariate analysis of variance (MANOVA) and Canonical variates analysis (CVA) utilizing the total specimen data set organized into 10 phylogenetically defined [34], [35], [36], [37], [38] family-level clades [groupings listed in Fig. 3]; (3) Euclidean and Neighbor-joining cluster analyses of all parameters averaged over these same families; and (4), a non-parametric one-way MANOVA (NPMANOVA), coupled with (5) a non-parametric one-way Analysis of similarities (ANOSIM), both manipulating the total data set divided into 14 stratigraphic source unit categories (collated from [39]). All calculations were conducted in *PAST* [40].

### (1) PCA, Bivariate Plots, and DA

**Figure 4:**
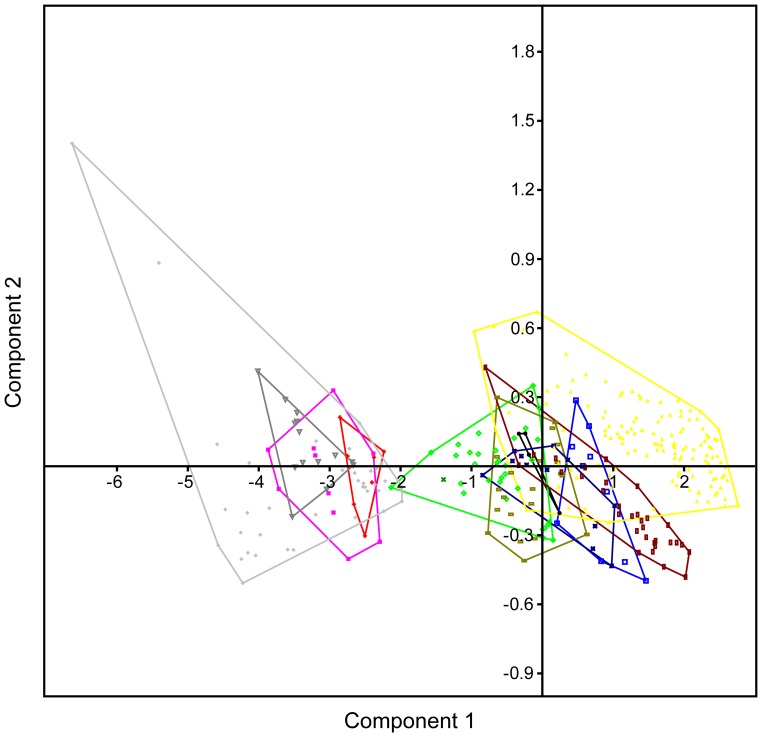
Principal components analysis plot of the most complete Adaffa Formation theropod tooth (SGS 0090) using CBL, CBW, CH, and AL. SGS 0090 (green x) is placed exclusively within the minimal scatter area (green polygon) of the sampled abelisaurid taxa, *Majungasaurus* and “*Indosuchus*”. Axis/symbol equivalencies shown in Fig. 3.

**Figure 5:**
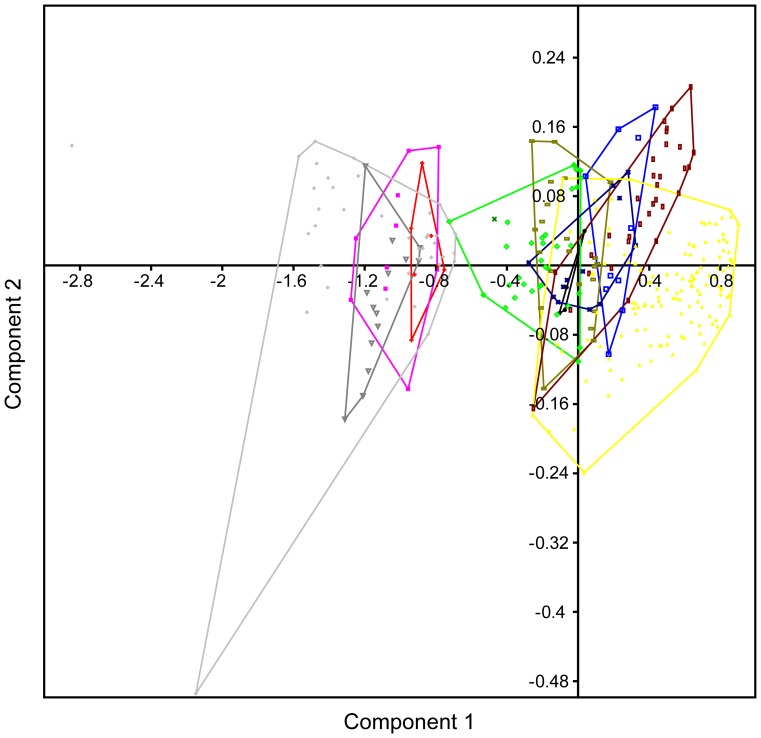
Principal components analysis plot of the most complete Adaffa Formation theropod tooth (SGS 0090) using CBL, CBW, and CH. SGS 0090 (green x) is placed exclusively within the minimal scatter area (green polygon) of the sampled abelisaurid taxa, *Majungasaurus* and “*Indosuchus*”. Axis/symbol equivalencies shown in Fig. 3.

**Figure 6:**
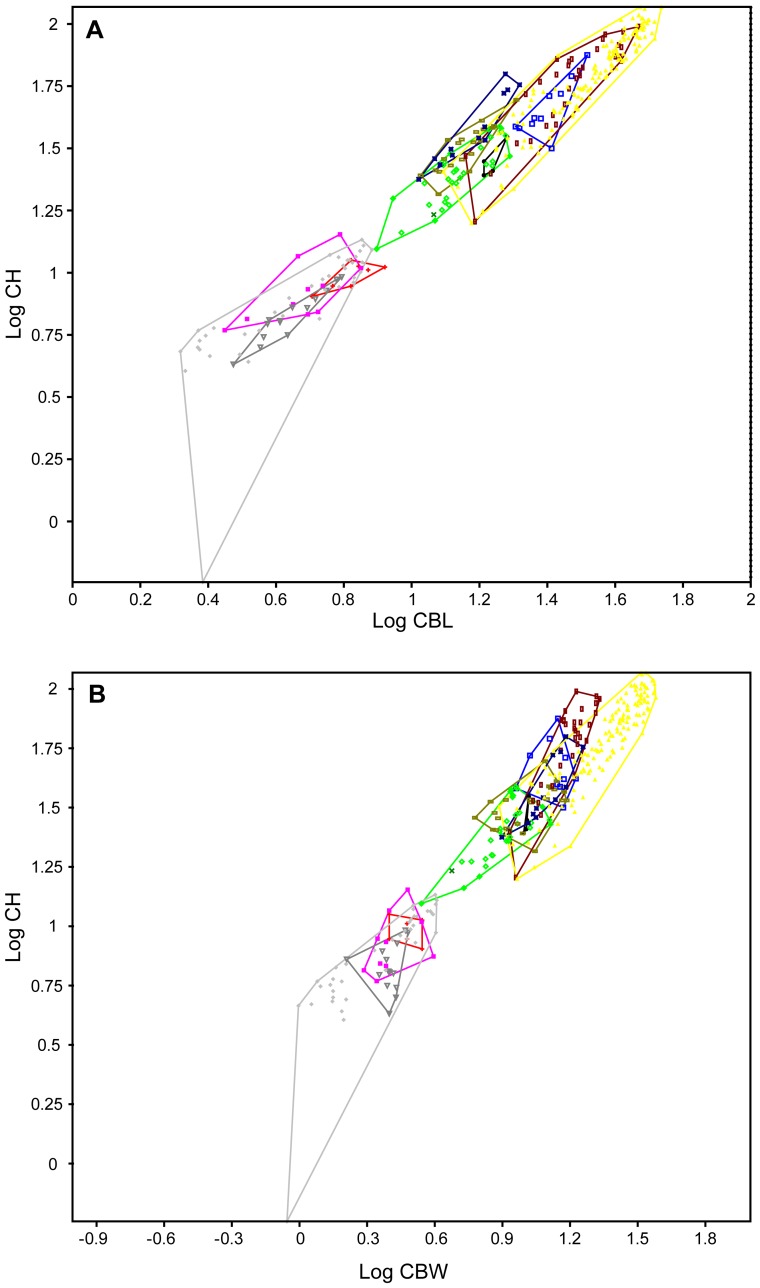
Bivariate plots of the most complete Adaffa Formation theropod tooth (SGS 0090) using CBL, CBW, and CH. Both A, CBL/CH; and B, CBW/CH tooth parameters indicate placement of SGS 0090 (green x) exclusively within the minimal scatter area (green polygon) of the sampled abelisaurid taxa, *Majungasaurus* and “*Indosuchus*”. Symbol equivalencies shown in Fig. 3.

We used a PCA, bivariate plots, and a DA to visualize the proposed linear relationship [41] between tooth base dimensions (CBL/CBW) and total crown height (CH), and to ascertain how this might affect our a priori shape-based classification of the most complete Adaffa Formation dinosaur tooth (SGS 0090). The PCA methodology followed Smith et al. [32] by incorporating CBL, CBW, CBR, CH, CHR, AL, CA, and DAVG (using a standard deviation normalized “correlation” setting for different units of measurement) to yield 67.869% of variance explained by the first principal component, and 13.666% by the second [scatter diagram in Fig. 3]. The first principal component is often thought to reify the size-determined shape vector [42], and in our analyses derived >0.96 of its correlated variable loading each from CBL, CBW, CH, and AL. Subsequent PCAs alternatively utilizing CBL, CBW, CH, and AL versus CBL, CBW, and CH, returned either 97.858% or 97.972% variance within the first principal component, and placed SGS 0090 exclusively amongst abelisaurids (*Majungasaurus* + “*Indosuchus*”) [Fig. 4, 5]. This implies close compatibility in their tooth base-height size parameters as depicted in bivariate plots of CBL/CH [Fig. 6A] and CBW/CH [Fig. 6B]; these also exhibited significant (RMA slope *a*  =  0.91227/*a*  =  1.0176) allometry as reported in other theropods [43]. Discriminant analyses of the same variables with an initial Box’s *M* test for homoscedasticity (raw data *p*<1.0223E-14; *p*<4.1647E-12), likewise classified SGS 0090 with abelisaurids. However, only 66.25%, or 65.63% respectively (a >90% hit ratio is normally considered distinct [44]), of teeth in the sample could be correctly identified, and there was no significant difference (*p*<0.001) in multivariate mean (Hotelling’s *T^2^*: *p*<0.3386; *p*<0.2171). Better compliance was achieved when all variables were examined collectively (as in previous studies [24], [32], [45]), with 80.94% correct classification. Nonetheless, there was still no significant difference between the multivariate means (Hotelling’s *T^2^*: p<6.725E-09), inferring that the variances were small in proportion to the distance between these two groups.

### (2) MANOVA and CVA

**Figure 7:**
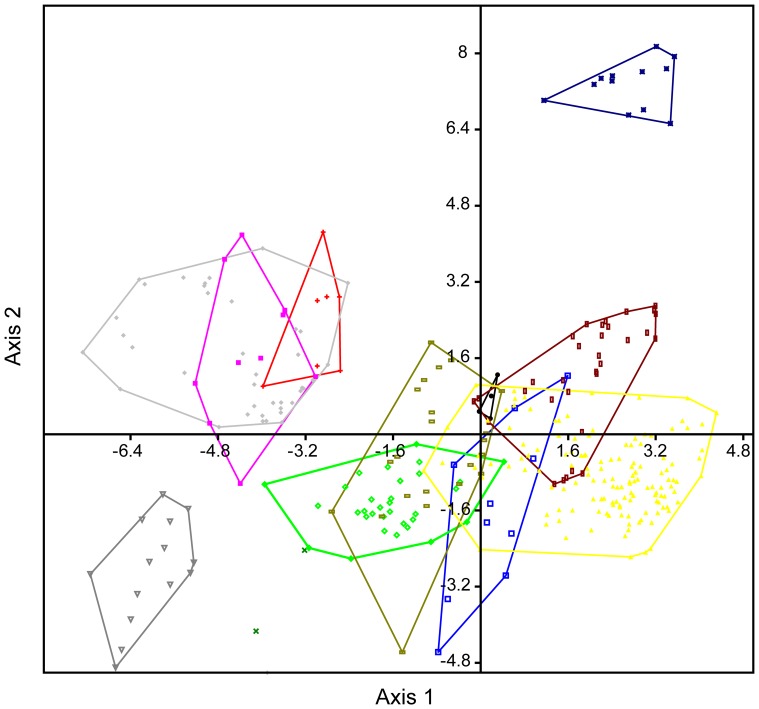
Canonical variate analysis plot of the Adaffa Formation theropod teeth (SGS 0061, SGS 0090) using all metric parameters. SGS 0061 + SGS 0090 represented by (green x); other symbol equivalencies shown in Fig. 3. Axes 1 and 2 depict maximum discrimination in the data set.

To further assess the equality of multivariate means (centroids) across multiple independent samples, and the impact of incorporating variables from both SGS 0090 and SGS 0061, we undertook a MANOVA with enforced Euclidean distance measure and depiction via a CVA [Fig. 7]. The MANOVA proceeded with pairwise comparisons between SGS 0090 + SGS 0061 and abelisaurids (*Majungasaurus* + “*Indosuchus*”), *Ceratosaurus*, *Masiakasaurus*, *Allosaurus*, carcharodontosaurids (*Acrocathosaurus* + *Carcharodontosaurus*), spinosaurids (*Baryonyx* + *Suchomimus*), tyrannosaurids (*Gorgosaurus* + *Daspletosaurus* + *Tyrannosaurus*), troodontids (*Troodon* + *Saurornithoides*), and dromaeosaurids (*Bambiraptor* + *Deinonychus* + *Dromaeosaurus* + *Velociraptor*). None of these taxa were found to be significantly different (*p*<0.05) from SGS 0090 + SGS 0061 in their tooth metrics. However, the minimum ( =  closest equality) squared Mahanalobis distance (*D^2^*) of 161.626 units was still returned between SGS 0090 + SGS 0061 and the *Majungasaurus* + “*Indosuchus*” centroid (Hotelling’s *T^2^*: *p*<6.53207E-11).

### (3) Cluster Analyses

**Figure 8:**
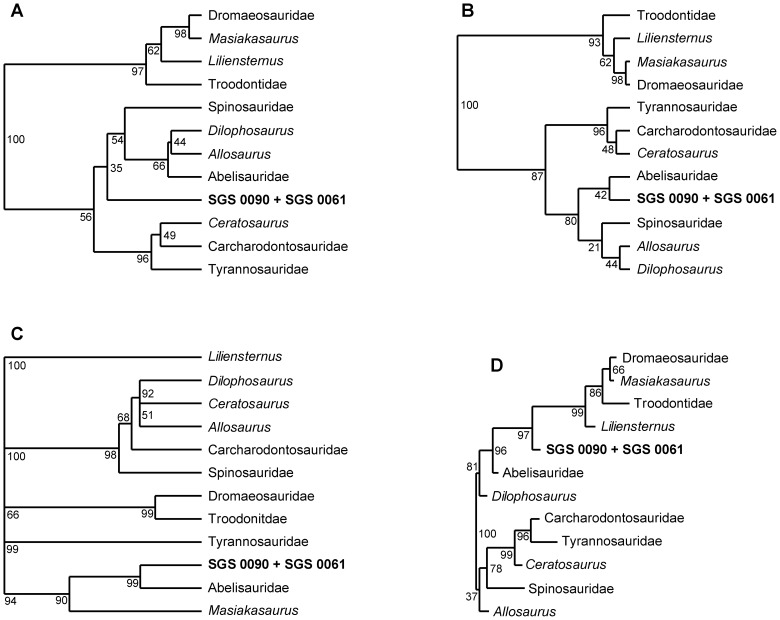
Dendrogram results of the cluster analyses. A, UPGMA; B, Ward’s method; C, chronostratigraphically constrained UPGMA clustering; D, phylogram generated by Neighbour-joining clustering. Node numbering represents bootstrap percentages from 1000 replicates.

We also employed a series of bootstrapped (1000 replicates) linear Euclidean cluster analyses to visualize the placement of SGS 0090 + SGS 0061 against a distance matrix of values averaged (so as to minimize sensitivity inherent in uneven sample sizes) over non-avian theropod family-level clades [see legend in Fig. 3]. Calculations used an unweighted paired-group average (UPGMA) [Fig. 8A] and Ward’s method, which joins clusters using minimal ingroup variance [Fig. 8B]. These approaches all derived unreliable support (< 50% bootstrap values); however, Larson and Currie [46] recently reported that metrically compatible Late Cretaceous theropod teeth display marked source unit specificity. We therefore chronostratigraphically constrained our UPGMA to follow stage-level time bins (with temporal ranges for family-level taxa defined using recognized phylogenetic boundaries [34], [35], [36], [37], [38]), and subsequently derived a much more robust SGS 0090 + SGS 0061 + abelisaurid grouping (99% bootstrap value [Fig. 8C]). Nevertheless, cross-correlation with Neighbour-joining again failed to generate comparable agglomerations when using stratigraphically unconstrained data [see Fig. 8D].

### (4) NPMANOVA

To alternatively examine SGS 0090 + SGS 0061 as a locality sample, rather that simply assume taxonomic homogeneity, we differentiated multivariate data points over the entire distance matrix with taxa grouped by their stratigraphic source units. This employed a NPMANOVA with default Bray-Curtis, as well as user specified Euclidean distance measures. Significant difference (*p*<0.05) was found between SGS 0090 + SGS 0061 and *Liliensternus*, *Ceratosaurus*, *Masiakasaurus*, *Allosaurus*, *Acrocathosaurus*, *Carcharodontosaurus*, *Baryonyx*, *Gorgosaurus*, *Daspletosaurus*, *Tyrannosaurus*, *Troodon*, *Saurornithoides*, *Bambiraptor*, *Deinonychus*, and *Dromaeosaurus*. A minimum *F* statistic value ( =  closest distance) of 0.4586 (Bray-Curtis)/0.5882 (Euclidean) was found for SGS 0090 + SGS 0061 and the derived carnotaurine *Majungasaurus*, with a strong probability of equality from 10000 permutations (*p*<0.6106; *p*<0.559).

### (4) ANOSIM

As a cross-correlation for the NPMANOVA results, we also calculated an ANOSIM (using Bray-Curtis and Euclidean distance measures) to compare distances both within and between our designated stratigraphic groupings. This derived significant distance (*p*<0.05) between SGS 0090 + SGS 0061 and *Liliensternus*, *Ceratosaurus*, *Masiakasaurus*, *Allosaurus*, *Acrocathosaurus*, *Carcharodontosaurus*, *Baryonyx*, *Gorgosaurus*, *Daspletosaurus*, *Tyrannosaurus*, *Troodon*, *Saurornithoides*, *Bambiraptor*, *Deinonychus*, and *Dromaeosaurus*. Closest approximation occurred between SGS 0090 + SGS 0061 and abelisaurids, with the minimum positive *R* value ( =  least distance) of 0.02938 (Bray-Curtis)/0.0853 (Euclidean) again indicating nearest placement to *Majungasaurus* (probability of equality from 10000 permutations: *p*<0.3756; *p*<0.2829).

## Conclusions

Despite being fragmentary, the Adaffa Formation dinosaur remains are justifiably referable to well-known Late Cretaceous taxa: Titanosauria, the globally dominant group of Campanian-Maastrichtian sauropods [47], robustly diagnosed by the presence of procoelous caudal centra [29] (alternatively this has been considered a synapomorphy for the constituent clade Lithostrotia [21]); and Abelisauridae, whose latest Cretaceous African distribution is substantiated by rare teeth [24] uniquely possessing a centrally positioned apex with strongly curved mesial, and straight distal profiles [30]. Closely compatible dental morphometry derived using an established data set [24], [32], [45] and methodologies [33], [41], [43], [46] contributes further support for this theropod classification.

The combined evidence of testable phylogentic character states and metric similarities, we believe, provides a rigorous basis for our taxonomic assignments, even when reliant upon a few incomplete specimens. This is important because dinosaur material from the Arabian Peninsula and Levant is otherwise limited to isolated traces, or rare assemblages recognized only from non-diagnostic body fossils [3], [4] and track ways [11]. Given this dearth of co-occurring remains, nothing has yet been gleaned of Arabian dinosaur diversity other than the sympatric presence of indeterminate ornithopods, sauropods, and theropods during the Maastrichtian [3], [4]. The recovery of demonstrably coeval titanosaurian (possibly lithostrotian) and derived abelisaurid remains in the Adaffa Formation of Saudi Arabia therefore provides the first taxonomic verification of faunal composition within the region. Moreover, it brings to light the only definitively identifiable example of a non-avian theropod dinosaur clade from the Arabian subcontinent, and one that shows closest compatibility with penecontemporaneous faunas in Africa [24] and Madagascar [23].

The Arabian Peninsula was contiguous with the main North African landmass during the Late Cretaceous [see Fig. 1], and would have experienced uniform equatorial climates and vegetational regimes [18]. Phylogenetic coherence of the Adaffa Formation dinosaur remains with quintessential northern Gondwanan faunal elements is therefore not surprising. However, the Afro-Arabian record of titanosaurians and abelisaurids is not only extremely poor, but also mainly restricted to the pre-Cenomanian Cretaceous [48], [30]. Thus the Saudi Arabian fossils, together other finds from the latest Cretaceous (Maastrichtian) of Morocco [49], [50], Jordan [2], and Egypt [24], provide important evidence of the palaeogeographical ubiquity of these taxa along the northern Gondwanan margin towards the end of the Mesozoic.
